# *Apolipoprotein* *B48* Knockout Ameliorates High-Fat-Diet-Induced Metabolic Impairment in Mice

**DOI:** 10.3390/biom15101454

**Published:** 2025-10-15

**Authors:** Yale Tang, Chao Wang, Luxuan Li, Xiaoyu Wang, Linquan Yang, Xing Wang, Luping Ren, Guangyao Song

**Affiliations:** 1 Department of Internal Medicine, Hebei Medical University, Shijiazhuang 050017, China; 23032100224@stu.hebmu.edu.cn (Y.T.);; 2 Department of Endocrinology, Hebei General Hospital, Shijiazhuang 050051, China; 3 Hebei Key Laboratory of Metabolic Diseases, Hebei General Hospital, Shijiazhuang 050051, China

**Keywords:** knockout, hyperlipidaemia, metabolism

## Abstract

This study aimed to investigate whether knockout of the *ApoB48* gene improves lipid metabolism disorders induced by a high-fat diet (HFD) in mice. Clustered regularly interspaced short palindromic repeats–Cas9 gene editing technology was used to knock out the *ApoB48* gene in C57BL/6J mice, and genotype identification showed heterozygosity (HE, *ApoB48* +/−). Subsequently, eight HE and eight wild-type (WT) mice were fed a HFD for 12 weeks. Fasting blood glucose, and insulin levels were decreased in *ApoB48* +/− mice. The intraperitoneal glucose tolerance test and intraperitoneal insulin tolerance test showed mild insulin resistance. Moreover, it delayed the development of atherosclerosis and intestinal tissue damage. Differential metabolites such as ceramide, sphingosine, and sphingosine-1-phosphate were identified using liquid chromatography–mass spectrometry, and differentially expressed proteins, including ceramide synthase 6 (CerS6), protein phosphatase 2A (PP2A), and protein kinase B (AKT), were indicated by the Kyoto Encyclopaedia of Genes and Genomes. Therefore, decreased expression of ApoB48 can ameliorate lipid metabolism disorders induced by an HFD, which may be related to the CerS6/PP2A/AKT pathway. This might represent a new approach for exploring methods to treat hyperlipidaemia.

## 1. Introduction

Hyperlipidaemia has become increasingly common due to lifestyle changes, with M=more than 25% of the population in Europe and the United States being affected [1,2]. In 2020, the prevalence of dyslipidaemia among young adults (aged 18–45 years) in the southeastern coastal region of China was 22.9% [3]. Hyperlipidaemia is a pathological condition characterised by lipid metabolism disorders caused by genetic susceptibility, dietary factors, environmental triggers, and metabolic disorders [4,5,6], which can lead to various harmful effects. Cohort studies have confirmed the correlation between hypertriglyceridaemia and atherosclerotic cardiovascular disease (ASCVD) [7]. Researchers predict that between 2010 and 2030, the number of cardiovascular events caused by hyperlipidaemia in China will increase to over 9 million [8]. Additionally, this condition can cause various metabolic disorders, such as acute and chronic pancreatitis, hyperglycaemia, and hepatic steatosis [9,10]. Therefore, the risks associated with metabolic complications pose a significant challenge to the prevention and treatment of hyperlipidaemia. Hence, exploring the underlying mechanisms is crucial. However, people spend most of their time in a postprandial state, and in clinical practice, and only fasting blood lipids are usually tested, meaning that postprandial hyperlipidaemia is often overlooked.

Furthermore, lipoproteins are closely associated with postprandial hyperlipidaemia and various metabolic disorders. Apolipoprotein B48 (ApoB48), a subtype of the lipoprotein B family, is a core apolipoprotein involved in exogenous lipid transport, that is, an intestinal lipoprotein. After food intake, dietary lipids are absorbed by small intestinal epithelial cells and subsequently assembled into chylomicrons in association with ApoB48. These chylomicrons transport lipids to peripheral tissues, especially adipose tissue, upon entering the systemic circulation. Abnormal function is directly related to chylomicron (CM) metabolic disorders and hypertriglyceridaemia. Each CM lipoprotein particle contains one ApoB48 molecule, and CM residues are thought to have multiple atherosclerotic properties [11]. Therefore, differences in *ApoB48* expression can cause metabolic disorders such as ASCVD. Previously, our research team and other researchers demonstrated a correlation between ApoB48 and metabolic disorders, such as postprandial hyperlipidaemia and non-alcoholic fatty liver disease [12,13], and postprandial hyperlipidaemia is also an important cause of ASCVD, which may represent a new target for treating hyperlipidaemia. In this study, we attempted to use a mouse model to reduce the expression of *ApoB48* and further investigate its mechanisms. This might help us to understand the lipid metabolism in patients with hyperlipidaemia and provide new ideas for the development of new drugs and clinical disease treatments.

The clustered regularly interspaced short palindromic repeat (CRISPR)–Cas9 system, composed of the Cas9 nuclease and modified single-conductor ribonucleic acid (sgRNA) [14], has been widely applied to the study of various diseases [15,16]. Gene-editing models such as *Apolipoprotein E* [17], *low-density lipoprotein receptor (LDLR)* [18], *Perilipin 1* [19], and *PCSK9* [20] have been successfully constructed to study lipid metabolism mechanisms. However, to date, there have been no reports of animal models with *ApoB48* knockout (KO).

Following *ApoB48* gene knockout, it is necessary to elucidate how it regulates metabolic disorders in hyperlipidaemia. Metabolomics, a biochemical method used to test dynamic changes in endogenous metabolites in biological systems, may be a highly suitable approach. Detection and analysis can provide objective and precise information on metabolic changes and disease conditions [21]. In recent years, metabolomics has been widely used to predict and diagnose various diseases, laying the foundation for discovering potential targets of clinical diseases [22]. Furthermore, metabolomics is a valuable method for studying biological lipids. Liquid chromatography–mass spectrometry (LC-MS) is the most widely used technique, offering good sensitivity and selectivity [23]. It can identify small molecule metabolites, the key substances reflecting physiological and pathological states [24] and important markers for early disease detection, which are helpful for exploring disease mechanisms.

This study aimed to successfully knock out the *ApoB48* gene in mice for the first time and feed them a high-fat diet (HFD) to explore the potential effects of improving hyperlipidaemia and regulating metabolic disorders, providing a theoretical basis for its potential application in the management of metabolic diseases.

## 2. Materials and Methods

### 2.1. Construction of Animal Models of Gene KO and Verification of Genotypes and Phenotypes

#### 2.1.1. The Design of sgRNA

**Figure 1:**
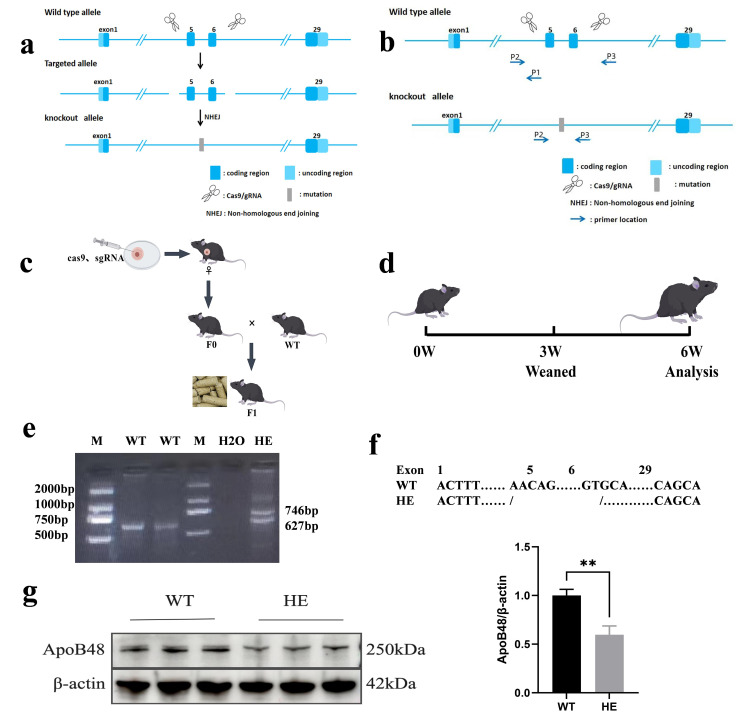
Generation of gene-edited mice. (**a**) Sequence strategy of exon 5–6 KO of the *ApoB48* gene based on CRISPR/Cas9. (**b**) PCR identification strategy after *ApoB48*. (**c**) Map of F1 generation KO mice; F0 gene-edited mice were mated with WT mice to obtain the F1 generation. (**d**) Test plan design. Mice were fed a normal diet until 6 weeks old. The initial phenotypic analysis verified the success of the model. (**e**) PCR analysis was performed using the tail genomic DNA. A single 627 bp band indicates the WT genotype, and two bands (746 bp and 627 bp) indicate the HE genotype. (**f**) Comparison chart of KO base pairs in the two groups. (**g**) Representative Western blot of intestinal ApoB48 in the indicated mice described in (**a**,**b**), and quantitative analysis of intestinal ApoB48 protein levels with different genotypes (*p* = 0.003, N = 3 in each group). All values are expressed as mean ± SD; ** denotes *p* < 0.01 HE vs. WT. Original Western blot images can be found in Supplementary Figure S4.

Basic information on target genes in the National Centre for Biotechnology Information. The CRISPR direct software was used to search for potential target sites (Supplementary Table S1), and the sgRNA in the line design tool was utilised to design the sgRNA for the exon 5–6 region of the *ApoB* gene (Supplementary Table S2). The mouse *ApoB* gene is located on chromosome 12, and a KO strategy diagram is shown in Figure 1a.

#### 2.1.2. Generation of Gene KO Mice

Human chorionic gonadotropin and pregnant mare serum gonadotropin were administered to induce superovulation in female C57BL/6J mice. Fertilised eggs were collected from mating cages containing male C57BL/6J mice of the same age. In vitro-transcribed sgRNA was combined with Cas9 protein and microinjected into the pronuclei of fertilised eggs. The injected embryos were transferred to pseudo-pregnant C57BL/6J mice. F0 generation mice were generated following parturition. Tailed deoxyribonucleic acid was extracted from the mice. Using Primer 6 software, two pairs of primers were designed to specifically target the upstream and downstream regions of the gene KO site, based on the sequences at both ends (Supplementary Table S3, Figure 1b). Genotypes were determined using polymerase chain reaction (PCR) amplification, agarose gel electrophoresis, and Sanger sequencing. The resulting F0 generation mice are chimeric and lack the capacity for stable genetic transmission due to the rapid early cleavage rate of fertilised eggs. Healthy F0 chimeric mice were selected and bred with wild-type (WT) mice of the opposite sex. Following parturition, F1 offspring exhibiting stable genetic integration (Figure 1c) were generated, and genotyping was performed using an identical methodology. At this point, the gene KO mice were generated.

#### 2.1.3. Experimental Animals

C57BL/6J mice were raised in Company Shanghai Nanfang Model Biological Technology Co., Ltd. (licence number: SCXK, Shanghai, China, 2019–0002). They were the offspring generated by crossing F1 generation gene KO with WT mice. In this experiment, both the WT mice and the gene KO mice were descendants of F1. They were weaned at 3 weeks of age, housed separately by sex, and maintained at the Experimental Animal Centre of Hebei General Hospital. The mice were fed in a specific pathogen-free room and housed in an air-conditioned room with a 12/12 h light/dark cycle, at a temperature of (24 ± 2) °C and relative humidity of (60 ± 10)%, with 8–10 air changes/h. Further details are provided in Supplementary Table S1.

#### 2.1.4. Biochemical Indicators Measurement

Overall, 45 WT mice (males N = 30, females N = 15) and 42 HE mice (males N = 30, females N = 12) were fed a normal diet until age 6 weeks (Figure 1d) and weighed; tail blood was collected after fasting for 12 h. Triglycerides (TGs) were measured using the glycerol phosphate oxidase–peroxidase method; total cholesterol (TC) was measured using the cholesterol oxidase–peroxidase coupled method; and high-density lipoprotein cholesterol (HDL-C) and low-density lipoprotein cholesterol (LDL-C) were assessed using the direct two-reagent method. Non-HDL-C = TC − HDL-C and triglyceride-rich lipoproteins (TRLRs) = TC − HDL-C − LDL-C. Fasting blood glucose (FBG) was measured using a blood glucometer.

#### 2.1.5. Histopathologic Evaluation

Two male mice were randomly dissected from each group and stained with haematoxylin & eosin (H&E) to observe the pathological changes in the entire intestinal tissue.

### 2.2. HFD Intervention

A successful establishment of the gene KO model was demonstrated using simple phenotypic analysis (Supplementary Table S4). To further explore whether KO of ApoB48 can ameliorate high-fat-diet-induced metabolic impairment in mice, male C57BL/6J mice (N = 8 gene-edited mice, N = 8 WT mice) were randomly selected and fed a HFD for 12 weeks.

#### 2.2.1. Intraperitoneal Glucose Tolerance Test (IPGTT) and Intraperitoneal Insulin Tolerance Test (IPITT)

At the 11th week of HFD feeding, all mice underwent an IPGTT. The mice were fasted for 12–14 h before the test and intraperitoneally injected with glucose (2 g/kg). Blood was collected from the tails of mice at 0, 15, 30, 60, and 120 min to measure blood glucose levels. A similar method was applied to perform the IPITT at the end of week 12. After fasting for 4–6 h, the mice were injected intraperitoneally with glucose (0.5 IU/kg). Blood glucose levels were measured at 0, 15, 30, 60, and 120 min.

#### 2.2.2. Biochemical Indicators Measurement

After fasting for 12 h, blood was collected from the internal canthal vein under anaesthesia, followed by dissection. Lipids were measured using the same kit as described earlier. Fasting insulin (FINS) and ApoB48 were detected using an enzyme-linked immunosorbent assay kit; FBG levels were also measured. Homeostasis model assessment for insulin resistance (HOMA-IR) = FPG (mmol/L) × FINS (μU/mL)/22.5 and quantitative insulin sensitivity check index (QUICKI) = 1/(Ig FINS + Ig FPG) were used to assess insulin resistance and sensitivity.

#### 2.2.3. Collection and Staining of Tissue Samples

After complete anaesthesia, the mice were dissected, and the tissues were separated and weighed. The tissue-to-body ratios were calculated. The lipid content in the intestinal and fat tissues was measured. The intestinal tissue and aorta were stained with H&E and oil red O (ORO) to observe the lipid deposition. Moreover, the aortic sinus was stained with Masson’s trichrome to analyse the degree of tissue fibrosis.

To assess lipid deposition in the entire aorta or in individual regions, and based on previous reports [25], we defined four segments as follows: (1) aortic arch, from the aortic root to 3 mm below the left subclavian; (2) thoracic aorta, the region between the end of the arch and the last intercostal branch; (3) abdominal aorta, the region between the end of the thoracic aorta segment and the iliac bifurcation; and (4) iliac arteries, the portion below the iliac bifurcation.

### 2.3. Metabolic Profiling

In total, 50 μL of the serum sample was used for LC-MS analysis. The metabolites were annotated using the Kyoto Encyclopaedia of Genes and Genomes (KEGG) and the Human Metabolome Database. Multivariate statistical analyses, including orthogonal partial least squares discriminant analysis (OPLS-DA) and principal component analysis (PCA), were performed to observe metabolic differences. Metabolites with significant differences were identified based on variable importance in projection (VIP) > 1 and *p* < 0.05 (*t*-test, volcano plot, and heat map), and differentially expressed proteins were analysed using KEGG.

The levels of differentially expressed metabolites were detected using an assay kit, and Western blot analysis was used to determine the expression levels of relevant proteins in the differentially expressed pathways. Image J software (version 1.52a) was used to determine the grey values of the target bands and perform statistical analyses.

### 2.4. Western Blot

Based on the metabolomics results, a differentially regulated metabolic pathway was identified, and the associated protein expression levels were further analysed using Western blot. An appropriate amount of intestinal tissue was taken from mice and ground using a cryogenic grinder. Protein concentration was determined using the BCA method. Subsequently, an SDS-PAGE gel was prepared, protein samples were loaded into the wells, and perform electrophoresis was performed. Proteins were then transferred onto a PVDF membrane. The PVDF membrane was incubated overnight at 4 °C with the primary antibody diluted according to the recommended proportion. The next day, after washing the PVDF membrane three times, it was incubated with horseradish peroxidase conjugated secondary antibody and imaged by chemiluminescence. Detailed information on reagents and antibodies is provided in Supplementary Table S1.

### 2.5. Statistical Analysis

GraphPad Prism 9.0 was used for graphing. Data were presented as mean ± standard deviation and analysed using IBM SPSS Statistics software 26.0. Parameters between the two groups were compared using Student’s *t*-test. The value of *p* < 0.05 was considered statistically significant.

## 3. Results

### 3.1. Construction of Gene KO Mice Models and Phenotypic Detection

#### 3.1.1. Successful Construction of Gene KO Mice Using CRISPR/Cas9 Technology

F0-generation mice were crossed with WT mice to produce F1-generation mice. Mouse tail genotype identification was performed using PCR and agarose gel electrophoresis (Figure 1e). Four F1 mice were confirmed to be genotype heterozygous (HE) with a deletion of 2283 base pairs in the target region (Figure 1f), and Sanger sequencing results showed a double peak (Supplementary Figure S1).

The F1 generation was further bred, and the average litter size was similar to that of WT mice, indicating that *ApoB48* KO does not affect the reproductive capacity of the mice. Western blotting showed that the expression level of the ApoB48 protein was significantly decreased, confirming the successful construction of gene-edited animals (Figure 1g and Supplementary Figure S4).

#### 3.1.2. Serum Biochemical Indicator (6 Weeks Old)

The mice in groups HE and WT were raised under normal dietary conditions until 6 weeks of age. Visual inspection revealed that both groups had intact and glossy fur with no significant differences in body length. No noticeable differences were observed in the serum colour or transparency between HE and WT (Supplementary Figure S2). The TG, TC, and LDL-C levels decreased by 28.00%, 6.26%, and 46.58%, respectively. Notably, HDL-C levels also decreased. TRLRs, non-HDL, FBG, and body weight were also relatively lower in the HE group; however, the differences were not statistically significant (Supplementary Table S4).

Furthermore, we investigated whether the lipid levels in the animals were related to sex. Under the same environmental conditions as described earlier, no obvious differences were observed in serum colour and transparency between the groups, and no sex differences were observed in blood lipids, FBG, and body weight for the same genotype (Supplementary Table S5).

#### 3.1.3. Histopathologic Evaluation

After H&E staining of the intestinal tissue, both groups exhibited normal structures, characterised by good villus morphology and no obvious epithelial cell shedding. However, occasional fat droplets were observed in the WT group, whereas almost no fat droplet deposits were observed in the HE group (Supplementary Figure S2).

### 3.2. ApoB48+/− Can Mitigate the Harmful Effects of HFD

Eight male WT mice and eight male HE mice were randomly selected and fed an HFD for 12 weeks; the two groups were designated as HF-WT and HF-HE, respectively.

#### 3.2.1. Comparison of Appearance, Body Weight, and Food Intake Between the HF-WT and HF-HE Groups

**Figure 2:**
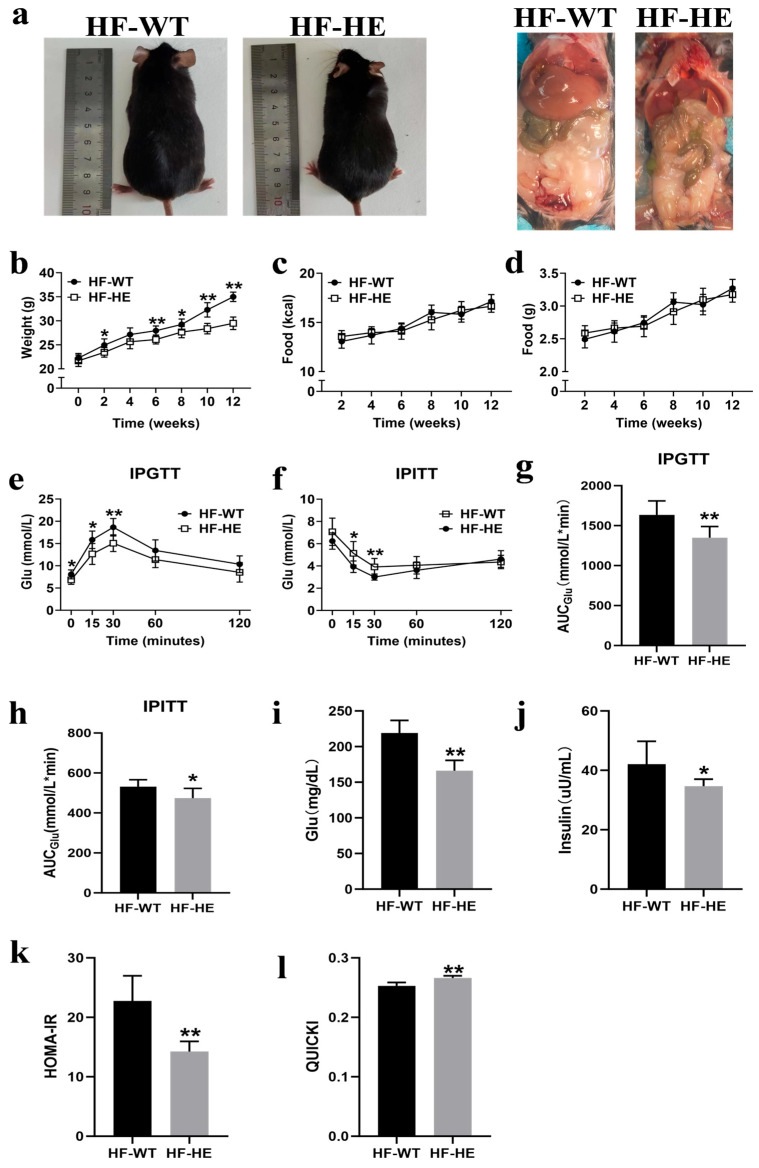
Comparison of body weight and glucose between the HF-WT and HF-HE groups. (**a**) Gross morphology and appearance after dissection of mice following HFD. (**b**) Trends in body weight in the HF-WT and HF-HE groups over 12 weeks of HFD, with significant differences beginning in the 6th week (N = 8 in each group, in the 6th week: *p* = 0.002). (**c**,**d**) Average daily energy intake and daily food intake (N = 8 in each group). (**e**) Blood glucose levels at 0, 15, 30, 60, 120 min during IPGTT (N = 8 in each group, 0 min: *p* = 0.026, 15 min: *p* = 0.011, 30 min: *p* = 0.002, 60 min: *p* = 0.069, 120 min: *p* = 0.100). (**f**) Blood glucose levels at 0, 15, 30, 60, 120 min during IPITT (N = 8 in each group, 0 min: *p* = 0.120, 15 min: *p* = 0.012, 30 min: *p* = 0.006, 60 min: *p* = 0.258, 120 min: *p* = 0.434). (**g**,**h**) 2 h AUC_Glu_ area under the curve of glucose (N = 8 in each group, *p* = 0.003, *p* = 0.017). (**i**,**j**) Fasting blood glucose and fasting blood insulin after 12 weeks of HFD (N = 8 in each group, *p* < 0.001, *p* = 0.020). (**k**,**l**) HOMA-IR and QUICKI of mice fed the HFD for 12 weeks (N = 8 in each group, *p* < 0.001, *p* < 0.001). Different labels over bars denote a significant difference. * *p* < 0.05, ** *p* < 0.01, compared with the HF-WT group.

No difference was observed in body length between the two groups. However, the mice in the HF-WT group had a rounded body shape. After dissection, they exhibited higher fat content, lighter liver colour (greyish-brown), and an oily lustre on the surface (Figure 2a). During the HFD period, the body weights of both groups showed an upward trend, with the HF-WT group demonstrating a higher body weight than the HF-HE group. After 12 weeks, the body weight of mice in the HF-WT group was 20% higher than that of the mice in the group (Figure 2b). As the age of the mice increased, their food and calorie intakes gradually increased; nonetheless, no significant differences were observed in the HF-WT and HF-HE groups (Figure 2c,d).

#### 3.2.2. Comparison of Insulin Sensitivity and Resistance Between the HF-WT and HF-HE Groups

The IPGTT was performed at the 11th week. After the intraperitoneal injection of glucose, blood glucose levels increased gradually, reaching peak levels at 30 min in both groups. The blood glucose level of the HF-WT group was approximately 20% higher than that of the HF-HE group; however, it subsequently began to decline. Nevertheless, the blood glucose level of the HF-WT group was always higher than that of the HF-HE group, the speed of recovery to the physiological level was slower (Figure 2e), and the area under the curve (AUC_Glu_) was higher (*p* = 0.003) (Figure 2g), indicating that glucose tolerance was severely impaired.

The IPITT was performed at the 12th week. After the intraperitoneal injection of insulin, blood glucose levels gradually decreased. This downward trend in blood glucose levels was more pronounced in the HF-HE group. A significant difference was observed between the two groups at 30–60 min, after which time they gradually recovered. Nevertheless, throughout the process, blood glucose levels were always lower than the initial blood glucose levels (Figure 2f). The AUC_Glu_ value in the HF-WT group was higher than that in the HF-HE group (*p* = 0.017) (Figure 2h), indicating that insulin tolerance was more severely impaired.

After the HFD, mouse serum was collected to measure FBG and FINS levels (Figure 2i,j). In the HF-WT group, FBG and FINS were 1.32-fold and 1.21-fold those in the HF-HE group, respectively (*p* < 0.001, *p* = 0.020), with higher HOMA-IR and lower QUICKI, indicating milder insulin resistance in the HF-HE group (Figure 2k,l).

#### 3.2.3. Comparison of Lipid Levels Between HF-WT Group and HF-HE Group

**Figure 3:**
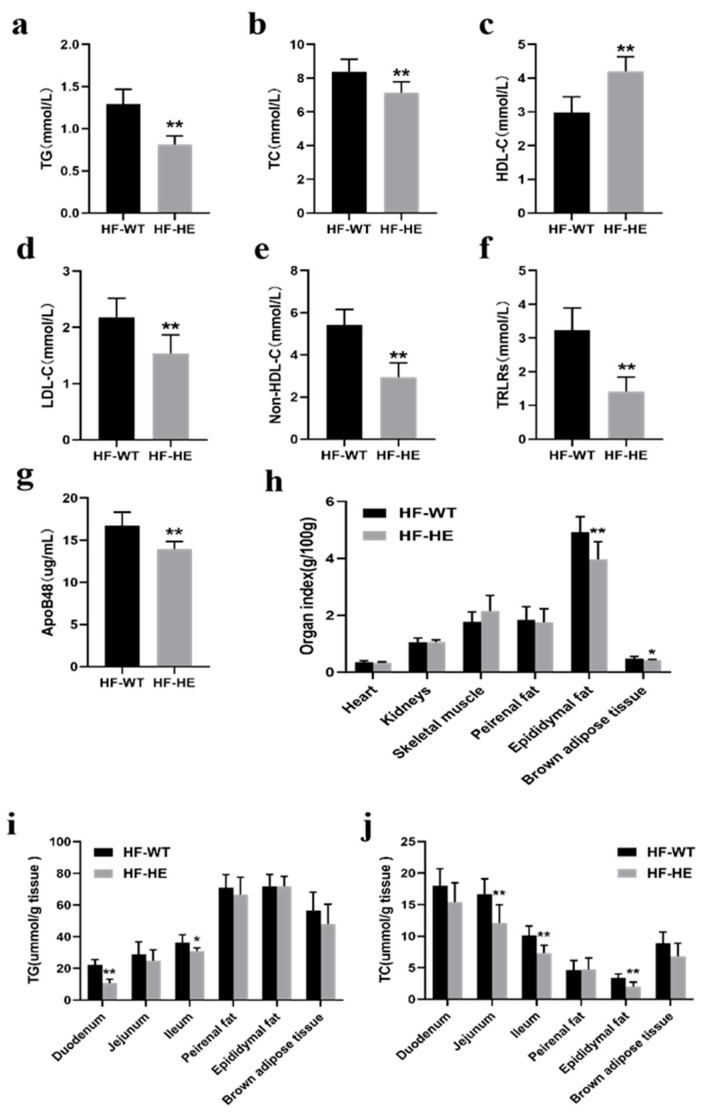
Lipid metabolism in the HF-WT and HF-HE groups. (**a**) TG (*p* = 0.003), (**b**) TC (*p* < 0.001), (**c**) HDL-C (*p* < 0.001), (**d**) LDL-C (*p* = 0.002), (**e**) non-HDL-C (*p* < 0.001), (**f**) TRLR (*p* < 0.001) levels in mouse serum (N = 8 in each group). (**g**) Serum ApoB48 level (*p* < 0.001, N = 8 in each group). (**h**) Heart (*p* = 0.752), kidney (*p* = 0.941), skeletal muscle (*p* = 0.116), perirenal fat (*p* = 0.726), epididymal fat (*p* = 0.006), and brown adipose tissue weight (*p* = 0.027) (N = 8 in each group). (**i**,**j**) TG and TC levels across tissues (N = 8 in each group). TG: duodenum *p* < 0.001, jejunum *p* = 0.294, ileum *p* = 0.012, epididymal fat *p* = 0.382, perirenal fat *p* = 0.978, brown adipose tissue *p* = 0.194. TC: duodenum *p* = 0.082, jejunum *p* = 0.004, ileum *p* = 0.001, epididymal fat *p* = 0.881, peirenal fat *p* = 0.001, brown adipose tissue *p* = 0.052. Different labels over bars denote a significant difference. * *p* < 0.05, ** *p* < 0.01, compared with HF-WT group.

The serum TG, TC, LDL-C, non-HDL-C, and TRLRs levels of mice in the HF-WT group were significantly higher than those in the HF-HE group, whereas the HDL-C levels were lower (*p* < 0.01). The serum level of ApoB48 in the HF-WT group was 19.94% higher than that in the HF-HE group (Figure 3a–g).

After dissection, the fat content in the HF-WT group was higher (Figure 2a). No significant difference was observed in the cardiac and renal indices; nevertheless, the weight of the skeletal muscle showed an increasing trend. The epididymal fat was significantly lower (*p* < 0.01). The perirenal adipose tissue demonstrated a decreasing trend, and the brown fat on the back diminished (Figure 3h).

After grinding the tissues and measuring the lipid content, the TG levels in the HF-WT group were lower, particularly in the duodenum and ileum. TC demonstrated the same trend, particularly in the jejunum, ileum, and epididymal fat (Figure 3i,j).

#### 3.2.4. Comparison of the Development of Atherosclerosis (AS) Between the HF-WT and HF-HE Groups

**Figure 4:**
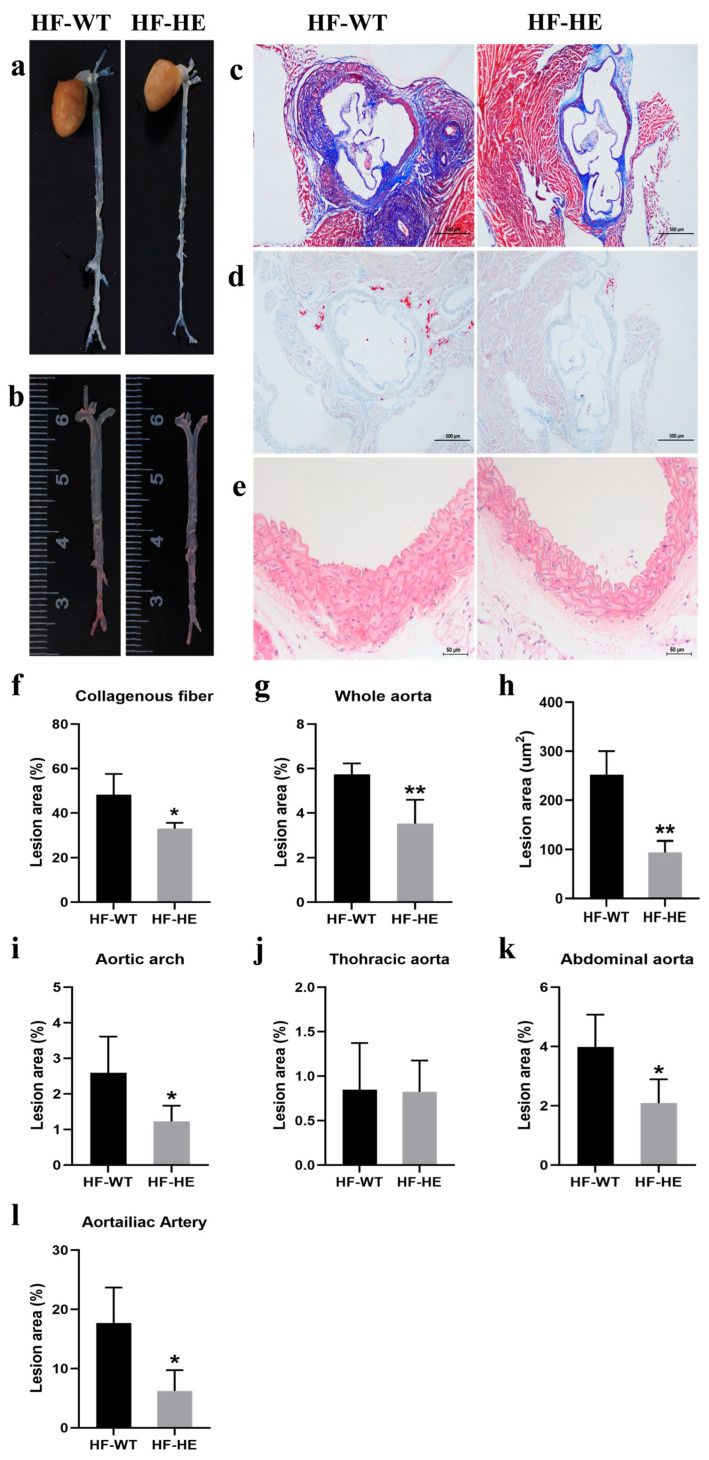
The development of AS. Representative images of (**a**) the en face whole aorta. (**b**,**g**) The ORO staining of the whole aorta with lesion areas quantified (*p* = 0.009, N = 4 in each group). (**c**,**f**) The Masson’s staining of the aortic root. (scale bar: 500 μm) with lesion areas quantified (*p* = 0.019, N = 4 in each group). (**d**,**h**) The ORO staining of the aortic root in the two groups with lesion areas are quantified (*p* = 0.001, N = 4 in each group). (**e**) The thickness of the vessel wall in the HF-HE (scale bar: 50 μm). Lesion sizes in the aortic arch (**i**), thoracic aorta (**j**), abdominal aorta (**k**) and aortailiac aorta (**l**) were quantified (*p* = 0.049, *p* = 0.945, *p* = 0.031, *p* = 0.016, N = 4 in each group). Different labels over bars denote significant differences. * *p* < 0.05, ** *p* < 0.01, compared with the HF-WT group.

The representative frontal image of the entire aorta after dissection and its length were relatively consistent (Figure 4a,b) at approximately 3–4 cm.

Masson staining of the aortic sinus showed that, in the HF-WT group, the overall arrangement of collagen fibres was more disordered (Figure 4c). The collagen fibre content in the HF-WT group reached 48%. In contrast, this was only 33% in the HF-HE group (*p* = 0.019) (Figure 4f). ORO staining also revealed significantly more red lipid droplets in the HF-WT group, approximately three times more than in the HF-HE group (Figure 4d,h). Vascular HE staining showed that the vascular wall thickness and extracellular matrix were relatively lower in the HF-HE group than in the HF-WT (Figure 4e). Differences were observed in the atherosclerotic plaques between the two groups, particularly in the abdominal and aortoiliac aortas (Figure 4g,i–l).

#### 3.2.5. Comparison of Intestinal Tissue Damage Between the HF-WT and HF-HE Groups

**Figure 5:**
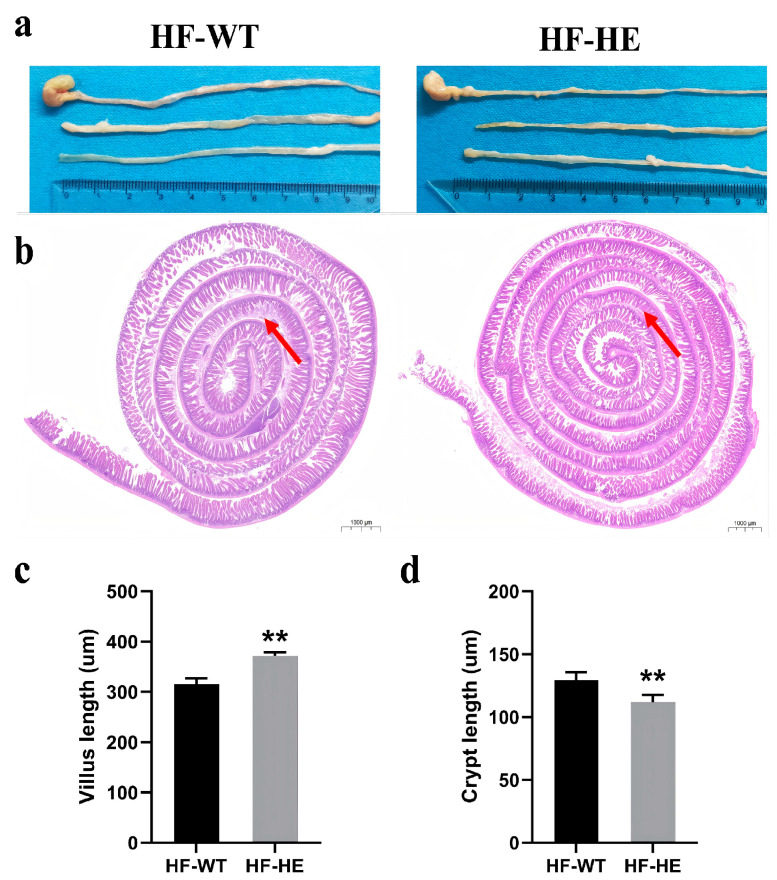
Intestinal tissue in the HF-WT and HF-HE groups. (**a**) Appearance and length in the HF-WT and HF-HE groups. (**b**) Entire intestinal tissue with HE staining. The position of the arrows indicated that in the HF-WT group, the arrangement of villi was more disordered and looser, the gaps increased, the loss was obvious, and the base of villi became shallower (scale bar: 1000 μm). (**c**,**d**) Villus length and crypt length in groups HF-WT and HF-HE. (*p* < 0.001, *p* = 0.007, N = 4 in each group). Different labels over bars denote a significant difference. ** *p* < 0.01, compared with the HF-WT group.

No significant differences were observed in the appearance and length of the intestinal tissues between HF-WT group and HF-HE group (Figure 5a). After staining, in the HF-WT group, the arrangement of villi was more disordered and looser, the gaps increased, an obvious loss was observed, the base of the villi narrowed (arrows, Figure 5b), the crypts became shallower, and the villus length increased (Figure 5c,d).

**Figure 6:**
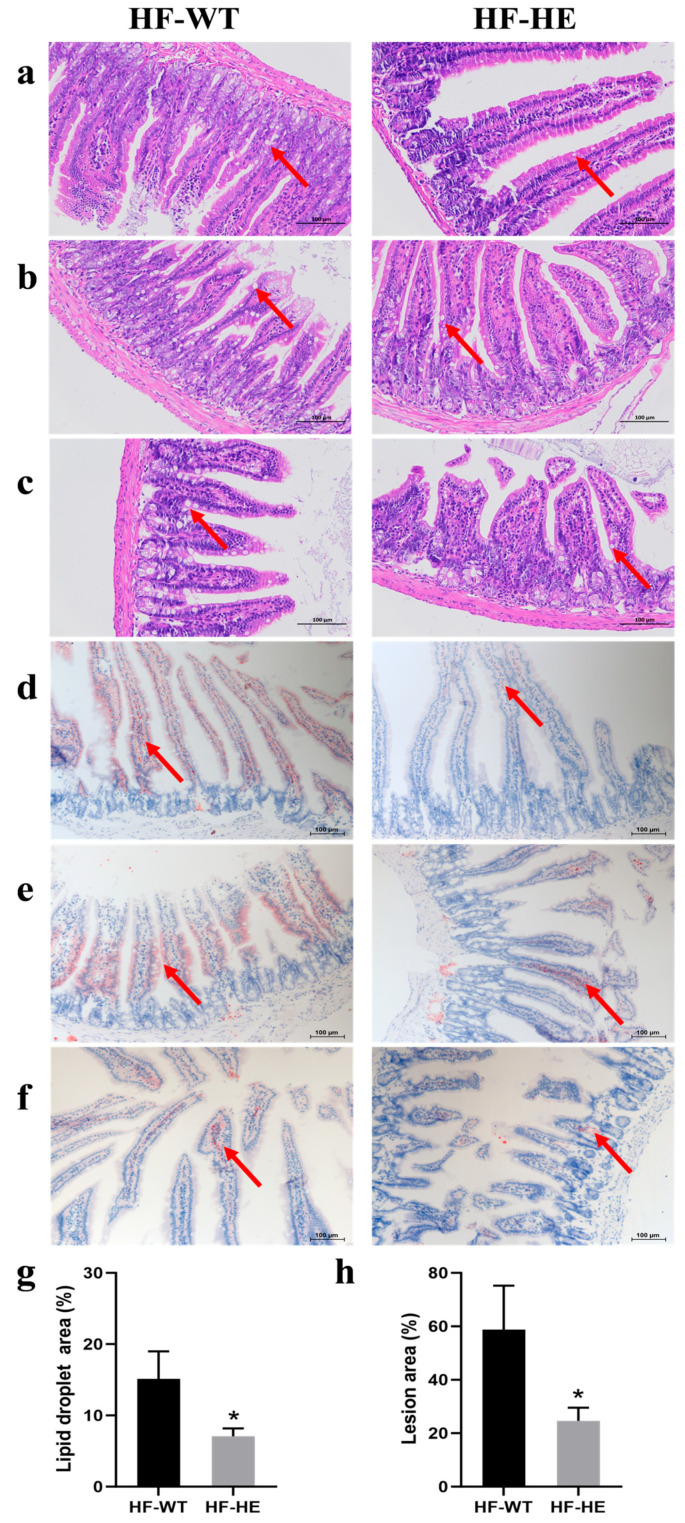
H&E and ORO staining in the HF-WT and HF-HE groups. H&E staining results of the duodenum (**a**), jejunum (**b**), and ileum (**c**). ORO staining results of the duodenum (**d**), jejunum (**e**) and ileum (**f**). The position of the arrows indicated the comparison of lipid droplets between groups HF-WT and HF-HE (scale bar: 100 μm). (**g**,**h**) Based on the staining results, the percentage of lipid droplet vacuole area under H&E and ORO staining to the total tissue area under the microscope in the above figure was statistically analysed (*p* = 0.027, *p* = 0.026, respectively). Different labels over bars denote significant differences. * *p* < 0.05,   compared with HF-WT group.

The small intestine was further stained in segments. H&E staining (Figure 6a–c) demonstrated that lipid droplet vacuoles (arrows) were more obvious in the HF-WT group than in the HF-HE group, especially in the jejunum and ileum (Figure 6b,c). The morphology of the intestinal villi in the HF-WT group was significantly abnormal, with some epithelial cell shedding. Increased inflammatory cell infiltration. ORO staining (Figure 6d–f) revealed that red lipid deposition was higher in the HF-WT group than in group HF-WE. The proportion of lipid droplet deposition was quantitatively analysed, and a significant difference was observed between the HF-WT group and the HF-HE group (Figure 6g–h).

### 3.3. Metabolic Analysis

#### 3.3.1. Quality Assessment of Serum Samples

Unsupervised PCA was used to eliminate intergroup interference, allowing for a clearer observation of intragroup changes. The original reaction data are presented in a scoring chart. OPLS-DA was also performed. The model is a supervised discriminant analysis statistical method that performs classification and prediction. It demonstrated that the classification is consistent with the PCA, indicating that significant differences are observed in the composition and structure of the metabolites. The metabolites near the upper right and lower left corners of the plot indicated a more significant difference. OPLS-DA verified that this model did not exhibit overfitting, indicating that the isolation of metabolites between groups was statistically significant (Supplementary Figure S3).

#### 3.3.2. Screen for Differential Metabolites

**Figure 7:**
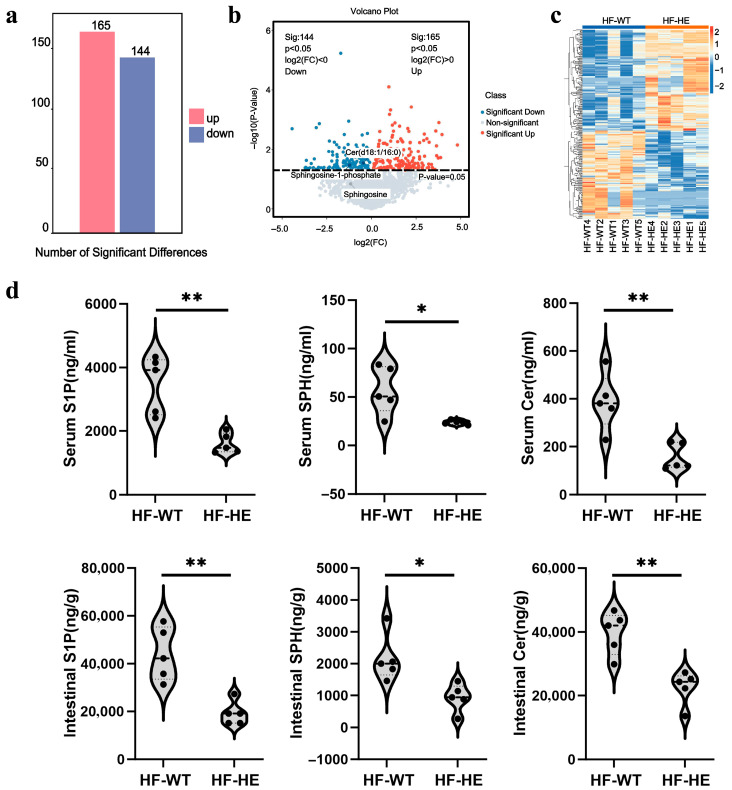
Quantitative and correlation analysis of differential metabolites. (**a**) Histogram of differential metabolites. (**b**) Volcano plot of spectral features with fold change > 1.5. (**c**) Hierarchical clustering. (**d**) Differences in metabolites in serum and intestinal tissues (*p* = 0.002, *p* = 0.017, *p* = 0.004, *p* = 0.002, *p* = 0.013, *p* = 0.002, N = 5 biologically independent experiments). All values are expressed as mean ± SD, * denotes *p* < 0.05 HF-HE vs. HF-WT, ** denotes *p* < 0.01 HF-HE vs. HF-WT. Parameters between the two groups were compared using Student’s *t*-test.

Overall, 3234 metabolites were identified, of which 309 showed significant differences, 165 were upregulated, and 144 were downregulated (Figure 7a). A volcano plot was used to assess statistical significance and to identify differences between the two groups (Figure 7b). Substances with similar expression patterns usually exhibit functional correlations across. Expression level data was used to calculate the clustering of substances or samples. To display the relationship between samples and the differences in metabolite expression among different samples more intuitively, the expression levels of all significantly different metabolites were subjected to hierarchical clustering graphs (Figure 7c), among which the expression level of ceramide d18:1/16 (Cer), sphingosine-1-phosphate (S1P) were significantly downregulated (Supplementary Table S6). The levels of S1P, sphingosine (SPH), and Cer in the serum and intestinal tissues also decreased by approximately 50% (Figure 7d).

#### 3.3.3. Differential Metabolic Pathways

**Figure 8:**
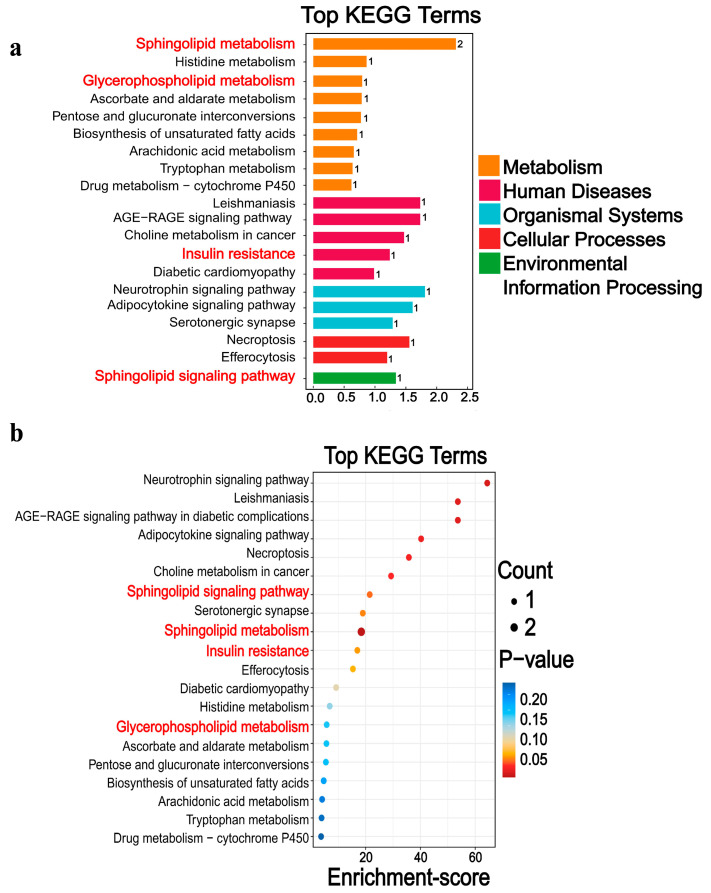
Differential metabolic pathways were screened by KEGG. (**a**) The 20 pathways with the lowest *p*-value. (**b**) The 20 pathways with the largest enrichment score. Differential metabolites are mainly involved in five broad categories. The main downregulation pathways include, among others, sphingolipid metabolism, insulin resistance, glycerophospholipid metabolism, and histidine metabolism. The red font denotes the relevant pathways of sphingomyelin metabolism.

**Figure 9:**
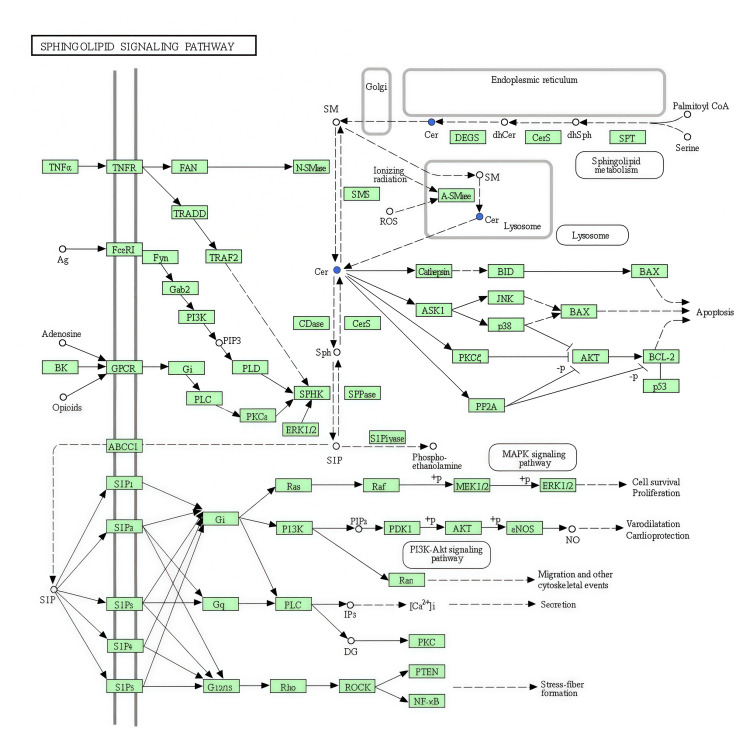
Sphingolipid signalling pathway in KEGG. The blue fonts represent the significantly downregulated representative differential metabolite Cer.

**Figure 10:**
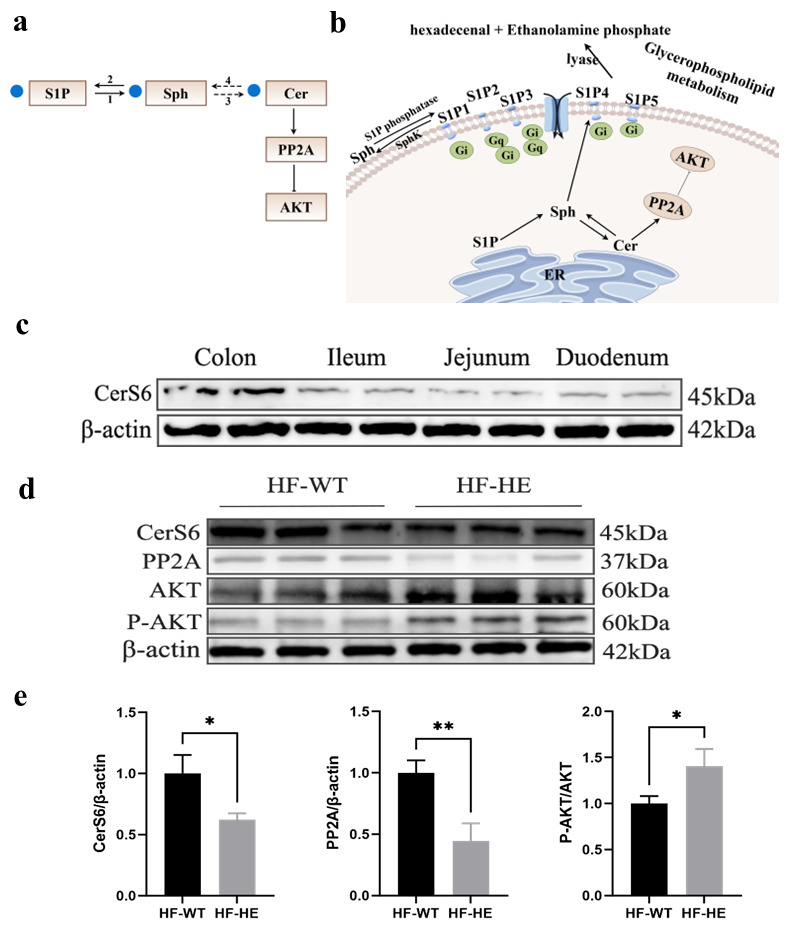
(**a**,**b**) Flow chart and simplified diagram of sphingolipid metabolism; blue dots represent significantly downregulated differential metabolites. S1P, sphingosine-1-phosphate; SPH, sphingosine; Cer, ceramide; PP2A, protein phosphatase-2A; 1, sphingosine-1-phosphate phosphatase; 2, SphK, sphingosine kinase; 3, ceramide synthase; 4, CDase, ceramidase. (**c**) The protein expression of CerS6 in different parts of the intestine in HF-HE (N = 2 in each tissue). (**d**,**e**) Changes in the expression levels of CerS6/PP2A/AKT signalling pathway proteins in the intestine (N = 3 in each group, CerS6: *p* = 0.015, PP2A: *p* = 0.005, AKT: *p* = 0.026). * denotes *p* < 0.05 HF-HE vs. HF-WT, ** denotes *p* < 0.01 HF-HE vs. HF-WT. Parameters between the two groups were compared using Student’s *t*-test. Original Western blot images can be found in Supplementary Figures S5 and S6.

The KEGG database displays molecular interaction networks, and 24 pathways were identified in the database. Both the *p*-value and the enrichment score indicated a significant association with the sphingomyelin metabolic pathway (Figure 8, Figure 9, Figure 10a,b and Supplementary Figures S5 and S6). To further verify the protein expression levels within this pathway, we assessed ceramide synthase 6 (CerS6) in different regions of the intestinal tissues of HF-HE mice (Figure 10c). Furthermore, the protein expression levels of the two groups were compared using intestinal tissues. The expression levels of CerS6 and protein phosphatase-2A (PP2A) were significantly decreased, whereas protein kinase B (AKT) was significantly phosphorylated, suggesting that the CerS6/PP2A/AKT pathway may participate in regulating lipid metabolism (Figure 10d,e).

## 4. Discussion

ApoB48 is synthesised in the rough endoplasmic reticulum (ER) and enters the ER lumen, where it combines with phospholipids, TC, and TG to form high-density CM particles. Each CM contains only one ApoB48 molecule, which remains in the CM throughout the blood circulation and metabolism processes and does not transfer to other lipoprotein particles. As the core protein for CM assembly, ApoB48 plays a central role in the exogenous lipid metabolism pathway and serves as an excellent surrogate marker for hyperlipidaemia. Multiple studies have shown that ApoB48 is associated with various metabolic diseases, such as kidney diseases, AS, and insulin resistance [26,27,28].

It is well known that two different forms of ApoB are formed in humans, ApoB48 and ApoB100. In this study, the KO strategy was designed to specifically abrogate ApoB48 production in the intestine. Therefore, it was not expected to directly affect hepatic ApoB100 expression at the genetic level. Nonetheless, the systemic metabolic consequences of ApoB48 deficiency could indirectly influence ApoB100 metabolism. Impaired assembly and secretion of chylomicrons due to the lack of ApoB48 leads to reduced dietary lipid absorption and lower postprandial lipidaemia. This improvement in the systemic lipid profile, as evidenced by decreased circulating lipid levels, may subsequently alleviate the metabolic burden on the liver. While our study demonstrates a clear protective role of *ApoB48* deletion against HFD-induced metabolic dysregulation, future investigations directly quantifying ApoB100 levels and kinetics would be invaluable to dissect the precise contributions of intestinal and hepatic ApoB pathways. Nonetheless, our findings strongly underscore the critical role of ApoB48-mediated lipid absorption in the occurrence of diet-induced metabolic syndrome. Furthermore, we performed an in-depth metabolomic analysis to explore the altered metabolic pathways.

After constructing the gene editing model, genotype detection was conducted first. Consistent with previous findings [29], where an interruption in the 5′ region of the *ApoB* gene led to mid-gestational embryonic lethality (before the 11.5th day of pregnancy) in homozygotes, our genotypic analysis confirmed that all successfully established models in this study were heterozygous (Figure 1e).

To verify the success of the model, a preliminary phenotypic analysis was conducted. In the HE group, TG, TC, and LDL-C levels decreased significantly, and a decreasing trend in non-HDL levels was also observed. Non-HDL refers to the total cholesterol contained in lipoproteins, except for HDL, in the blood, including cholesterol in very-low-density lipoprotein (VLDL), intermediate-density lipoprotein, LDL, and lipoprotein (a). It represents the total amount of cholesterol in *ApoB* lipoprotein particles, and this reduction may help lower plasma TC levels. Moreover, Voyiaziakis’s study [30] showed that TC levels increased sharply owing to an increase in non-HDL levels. Surprisingly, HDL-C levels in the HE group similarly decreased, which has also been documented in previous studies. Homanics et al. [31] synthesised a truncated form of *ApoB*, *ApoB70* by applying gene-targeting technology in mouse embryonic stem cells, which reduced the levels of *ApoB* messenger RNA in the intestines and livers of these mice. When plasma ApoB, TC, and TG levels decreased, a similar downward trend was observed in plasma HDL-C levels. Similarly, in the gene-edited mice constructed by ROBERT [29], the plasma TC level decreased by approximately 20% (81 ± 18 vs. 104 ± 17 mg/dL, *p* < 0.001), and the HDL-C level was also reduced by approximately 20%. Considering that particles carry most plasma cholesterol in mice without ApoB (HDL), the decrease in plasma TC can also be explained by the decrease in HDL-C. In humans and hamsters, when the TG level increases, it leads to an increase in the activity of the cholesterol ester transporter (CETP), causing an exchange between the core cholesterol ester of HDL-C and the core TG of VLDL-C, resulting in a decrease in HDL-C. However, CETP is absent in mice; therefore, conversion cannot occur, increasing HDL-C levels when TG levels increase.

Furthermore, when we examined whether the lipid levels in animals were related to sex, no significant sex differences were observed for the same genotype. Guo Xin et al. [25] used CRISPR/Cas9 technology to generate LDLR KO hamsters and obtained the same results, finding that no sex differences were observed in TC, TG, or HDL-C within the same genotype, indicating that no significant sex differences are observed in blood lipids between different genotypes (Supplementary Table S5).

Subsequently, the mice were fed a HFD for 12 weeks. A HFD can lead to abnormal blood lipid levels, insulin resistance, increased fat content, AS, tissue damage, and pathological changes [32], which have been confirmed in multiple HF mouse models [33,34,35,36]. Here, body weight was significantly decreased in the HF-HE group, with no changes in average daily energy intake or daily food intake (Figure 2b–d). Similar phenomena have been reported in multiple studies [37,38], with no difference in calorie intake after HFD; nevertheless, body weight decreased. In this study, the gene KO may have influenced the basal metabolic rate. The mice exhibited a heightened metabolic state, where the “expenditure” of energy exceeds the “intake”. Furthermore, at the end of the experiment, organ indices were measured, and the values were lower in the HF-HE group, indicating reduced lipid deposition in major organs, a finding corroborated during dissection. Additionally, we also observed an increase in fat content and serum lipids, along with plaque formation in AS in the HF-WT mice. The initiation of AS involves precisely the infiltration of lipids, LDL-C, and other lipoprotein cholesterol containing ApoB into the arterial wall, where macrophages phagocytose them to form foam cells, which accumulate to form lipid streaks and evolve into AS plaques [39]. Tian [12] and Nagao et al. [40] observed in clinical studies that the levels of ApoB48 and TG in patients with coronary artery disease or type 2 diabetes were significantly higher than those in healthy individuals. Therefore, lipoproteins containing ApoB48 may be involved in the development of AS [41]. A possible underlying mechanism is that *ApoB48* has binding sites for arterial wall proteoglycans [26]. Nonetheless, in HF-HE mice, these changes were significantly attenuated, and the progression of AS was delayed (Figure 4).

Through molecular level exploration, the differential metabolites were primarily enriched in sphingolipid metabolism and sphingolipid signalling pathways by LC-MS. Sphingolipids are the main lipid components of the eukaryotic plasma membrane and are bioactive lipid molecules. Sphingolipid metabolism plays a significant role in various processes, including insulin resistance and tumours [42,43,44]. In this study, the sphingolipid metabolism in group HF-HE was imbalanced (Figure 7). Sphingolipids are synthesised de novo from serine and palmitate in the ER and have a two-part structure, including a hydrophobic skeleton and a hydrophilic head group, which varies depending on the species. Sphingolipid metabolites primarily include long-chain-base SPH, long-chain-base 1-phosphate (namely sphingosine-1-phosphate receptors 1 through 5), and Cer. Cer is then decomposed by acidic CDases to form SPH [45]. SPH is a hydrophobic group that is almost insoluble in water and relatively neutral. Notably, it is found in all biological species, from bacteria to humans, participating in many enzymatic reactions and being crucial in the regulation of the actin cytoskeleton, endocytosis, cell cycle, and apoptosis. Additionally, SPH can block cholesterol transport by inhibiting LDL-C-induced cholesterol esterification, leading to the accumulation of unesterified cholesterol in perinuclear vesicles. Furthermore, it can be catalysed by sphingosine kinase 2 to produce S1P. As an intermediate in the sphingolipid–glycerophospholipid conversion, S1P is cleaved by S1P lyase into hexenal and ethanolamine phosphate, which enter the glycerophospholipid metabolic pathway (Figure 10b). Importantly, S1P lyase activity is particularly high in the small intestine. Therefore, in *ApoB*+/− mice, sphingolipid levels are reduced, and the conversion to glycerophospholipids in the small intestine is decreased, thereby affecting blood lipid levels.

Cer is composed of SPH (d18:1) long-chain bases and N-linked acyl chains [46] and is synthesised by ceramide synthase (CerS). To date, six CerS (CerS1–CerS6) have been identified, each with substrate specificity [47]. In this study, Cer was synthesised using the CerS6. Recently, the roles of CerS6 and its derivative Cer (d18:1/16:0) in metabolic diseases has attracted widespread attention. CerS6 inhibition significantly improves insulin resistance related to obesity [48,49]. Zhu et al. discovered that kidney diseases can be treated by targeting and regulating CerS6, which alleviates glomerular inflammation and podocyte damage in diabetic mice, and alters glycolipid metabolism [50]. This study suggests that the decreased level of Cer in the serum is due to the reduced expression of its upstream CerS6 protein, indicating that CerS6 is involved in lipid metabolism in lipoprotein gene-KO animal models, which may improve hyperlipidaemia (Figure 10c).

Excessive accumulation of Cer can activate PP2A, which is a major serine or threonine phosphatase composed of structural subunit A, regulatory subunit B, and catalytic subunit C. PP2A regulates numerous signal transduction pathways by counteracting most kinase-driven signalling pathways to maintain cellular homeostasis [51] and participates in the regulation of various diseases such as tumours, diabetes, heart disease, and human immunodeficiency virus [52,53]. Currently, a significant amount of research on tumours is being conducted, particularly regarding their role as tumour suppressor factors [54], while studies on lipid regulation are relatively scarce. Conversely, low concentrations of Cer may enhance insulin sensitivity and improve lipid distribution via PP2A. Some studies have shown that PP2A is involved in the excessive synthesis and deposition of TG in the liver [55], inducing the formation of high TG levels. Moreover, this study found that the protein expression level of PP2A, which participates in the regulation of lipid metabolism regulation, decreased in lipoprotein–KO animal models.

According to KEGG analysis, PP2A can cause the dephosphorylation and inactivation of AKT, promote fatty acid oxidation, and inhibit cholesterol synthesis. That is, when PP2A activity decreases, AKT undergoes phosphorylation. Notably, AKT acts as an intermediate signalling molecule, influencing glucose uptake and metabolism, regulating insulin sensitivity, and serving as a key mediator in insulin signalling and metabolic control, leading to deterioration of insulin responsiveness and islet cell death, and is closely associated with the development of metabolic syndrome. This process is referred to as lipotoxicity [56]. AKT is dephosphorylated under the regulation of phosphatase (mainly by regulating residue S473), leading to disorders in the expression of related regulatory proteins and signalling pathways. AKT is transferred from the cell membrane to the nucleus or cytoplasm when activated, altering the activity of target proteins through the phosphorylation and regulation of lipid synthesis. Furthermore, Sun et al. observed that lipogenesis and lipid accumulation can be inhibited by inducing AKT phosphorylation [57].

## 5. Conclusions

This study successfully established an *ApoB48* KO mouse model using CRISPR/Cas9 technology, which can improve various metabolic disorders caused by HFD, including increased fat content, hyperlipidaemia, insulin resistance, AS development, and intestinal tissue destruction. This phenomenon is related to the dynamic balance of sphingolipid metabolism. The levels of metabolites such as S1P, Cer, and SPH in the serum and tissue change, leading to the differential expression of related proteins. Considering that the CerS6/PP2A/AKT pathway is involved in lipid regulation, this study offers new insights into the treatment of hyperlipidaemia.

This study has some limitations. First, the sample size was too small to represent universal phenomena. In the future, we plan to conduct further research using a sufficient number of models to validate this view. Second, this study lacked in vitro experiments, which will be our next experimental plan. Third, the duration of the HFD can be appropriately extended, and the phenomenon of AS may become more obvious. Fourth, issues related to detection methods need to be considered, such as whether the measurement of metabolites is affected by blood lipid levels and intestinal contents or whether interference occurs during sample processing. For instance, haemolysis can cause red blood cells to release certain metabolites, potentially leading to errors in detection. In the future, multiple detection methods could be used to verify their reliability. Further research is needed to explore whether other regulatory pathways also play a role.

## Data Availability

The authors confirm that the data supporting the findings of this study are available within the article.

## Supplementary Materials

The following supporting information can be downloaded at: https://www.mdpi.com/article/10.3390/biom15101454/s1, Table S1: List of reagents; Table S2: sgRNA and PAM sequences; Table S3: The primers sequences; Table S4: The results of body weight and biochemical index detection of WT and HE groups; Table S5: Comparison of gender differences; Table S6: Differential metabolites; Figure S1: Sanger sequencing; Figure S2: Comparison of phenotypes; Figure S3: Overview and multivariate statistical analysis; Figure S4: Original Western blot images corresponding to Figure 1g in the main text; Figure S5: Original Western blot images corresponding to Figure 10c in the main text; Figure S6. Original Western blot images corresponding to Figure 10d in the main text.

## Abbreviations

Protein kinase B; AKT, atherosclerotic cardiovascular disease; ASVCD, area under the curve; AUC, ceramide synthase 6; CerS6, cholesterol ester transporter; CETP, chylomicron; CM, clustered regularly interspaced short palindromic repeat; CRISPR, fasting blood glucose; FBG, endoplasmic reticulum; ER, high-density lipoprotein cholesterol; HDL-C, heterozygous; HE, high-fat diet; HFD, homeostasis model assessment for insulin resistance; HOMA-IR, intermediate lipoprotein; IL, intraperitoneal glucose tolerance test; IPGTT, intraperitoneal insulin tolerance test; IPITT, Kyoto Encyclopaedia of Genes and Genomes; KEGG, liquid chromatography–mass spectrometry; LC-MS, low-density lipoprotein receptor; LDLR, low-density lipoprotein cholesterol; LDL-C, orthogonal partial least squares discriminant analysis; OPLS-DA, il red O; ORO, principal component analysis; PCA, polymerase chain reaction; PCR, protein phosphatase 2A; PP2A, quantitative insulin sensitivity check index; QUICKI, single-conductor ribonucleic acid; sgRNA, sphingosine; SPH, sphingosine-1-phosphate; S1P, total cholesterol; TC, triglycerides; TG, very-low-density lipoprotein; VLDL, wild-type; WT.
