# Supplementary material for: Apolipoprotein B48 Knockout Ameliorates High-Fat-Diet-Induced Metabolic Impairment in Mice
Source: Biomolecules. 2025 Oct 15;15(10):1454. doi: 10.3390/biom15101454 (PMC12564635; doi:10.3390/biom15101454)
Supplement: Supplementary file 1 [file biomolecules-15-01454-s001.zip › biomolecules-3892237-supplementary.pdf]

Supplementary Table S1 List of reagents

Reagents, instruments	Source	Details
Blood Glucose Meter	Roche Roccon , Germany	
Normal diet	Beijing Huafukang Biotechnology Co., LTD, Beijing, China. D12450J	10% energy from lipids, 70% energy from carbohydrates，20% energy from protein，providing 3.85kcal/g.
High-fat diet	Beijing Huafukang Biotechnology Co., LTD, Beijing, China. D12492	60% energy from lipids, 20% energy from carbohydrates, 20% energy from protein，providing 5.24kcal/g.
TG test kit	Jiancheng Bioengineering Institute, Nanjing, China.	Cat#: A110-1-1
TC test kit	Jiancheng Bioengineering Institute, Nanjing, China.	Cat#: A111-1-1
HDL-C test kit	Jiancheng Bioengineering Institute, Nanjing, China.	Cat#: A112-1-1
LDL-C test kit	Jiancheng Bioengineering Institute, Nanjing, China.	Cat#: A113-1-1
ApoB48 test kit	Meimian Industrial Co., Ltd, Jiangsu, China	Cat#: MM-45476M2
Insulin test kit	ALPCO, Shanghai, China	Cat#: 80-INSMSU-E01
BCA	Abbkine, Wuhan, China	
SDS-PAGE	Biotides, Beijing, China	
**Antibodies**		
Antibody β-actin	ZenBio (1:10000)	Cat#: 380624
Antibody CerS	ProMab (1:1000)	Cat#: Q6ZMG9
Antibody PP2A	ProMab (1:1000)	Cat#: P67775
Antibody AKT	Cell Signaling Technology (1:1000)	Cat#: 9272
Antibody P-AKT Ser473	Cell Signaling Technology (1:1000)	Cat#: 9271
Antibody ApoB48	Abcam (1:1000)	Cat#: AB312318
**Softwares**		
Graphpad prism 9.0	http:// graphpad.com	
Fiji Image J	http:// imagej.net/Fiji	
IBM SPSS 26.0	https://www.ibm.com/products/spss	
CRISPR	https://crispr.dbcls.jp/	
Primer design	https://www.premierbiosoft.com	
NCBI	https://www.ncbi.nlm.nih.gov	

Supplementary Table S2 sgRNA and PAM sequences

sgRNAs	Sequence (5′-3′)	PAM
sgRNA1	CAGTGATGCCCACAGAAACA	GGG
sgRNA2	TTCGAGGTATACATGTGCAT	GGG

Supplementary Table S3 The primers sequences

Name	Sequence (5′-3′)
**P2**	GCTAGAGACACGAGCTTCGG
P3	AGGCAGGAAAAGATGCTGCT
P1	GAAAAGCCATCCTTCCACTGAG

Supplementary Table S4 The results of body weight and biochemical index detection of WT and HE groups

	**[illegible] (Male, N = 30)**	**HE (Male, N = 30)**	**[illegible]**
TG (mmol/L)	1.00 ± 0.19	0.72 ± 0.20	< 0.001
TC (mmol/L)	5.91 ± 0.79	5.54 ± 0.48	0.03
HDL-C (mmol/L)	2.22 ± 0.52	1.97 ± 0.43	0.56
LDL-C (mmol/L)	1.46 ± 0.58	0.92 ± 0.30	< 0.01
Non-HDL (mmol/L)	3.70 ± 0.95	3.56 ± 0.57	0.51
TRLRs (mmol/L)	2.23 ± 1.06	2.64 ± 0.64	0.08
GLU (mmol/L)	5.9 ± 0.5	5.8 ± 0.5	0.26
Weight (g)	25.7 ± 0.8	25.3 ± 1.1	0.15

Supplementary Table S5 Comparison of gender differences

	**WT**		**P**	**HE**		**P**
	**Male (N = 30)**	**Female (N = 15)**		**Male (N = 30)**	**Female (N = 12)**	
TG (mmol/L)	1.00 ± 0.19	1.08 ± 0.16	0.15	0.72 ± 0.20	0.82 ± 0.11	0.09
TC (mmol/L)	5.91 ± 0.79	5.92 ± 0.46	0.96	5.54 ± 0.48	5.72 ± 0.36	0.36
GLU (mmol/L)	5.9 ± 0.5	5.8 ± 0.6	0.71	5.8 ± 0.5	5.7 ± 0.4	0.60
Weight (g)	25.7 ± 0.8	25.8 ± 0.7	0.72	25.3 ± 1.1	25.3 ± 0.6	0.93

Supplementary Table S6 Differential metabolites

Metabolites	Cer(d18:1/16:0)	Sphingosine-1-phosphate	Sphinganine-phosphate
Super Class	Lipids and lipid-like molecules		
Score	38.1	68.7	69.5
Average (HF-HE)	12.86507362	15.794919	18.008933
Average (HF-WT)	13.31606494	17.015581	18.693905
log2FoldChange	-0.45099132	-1.220662	-0.684972
FoldChange	0.731540011	0.4290859	0.6220179
Regulation	Down	Down	Down
p-value	0.028777618	0.0338649	0.0774595
HF-HE1	12.84588605	15.828542	18.100507
HF-HE2	12.640641	16.222888	18.281418
HF-HE3	12.87746569	15.281387	17.468927
HF-HE4	13.17347184	15.973259	18.099075
HF-HE5	12.78790351	15.668521	18.094739
HF-WT1	12.94036754	16.221132	18.121709
HF-WT2	13.27993089	17.980038	19.472166
HF-WT3	13.12784211	18.142588	19.399058
HF-WT4	13.79605547	16.810674	18.401409
HF-WT5	13.43612868	15.923473	18.075183

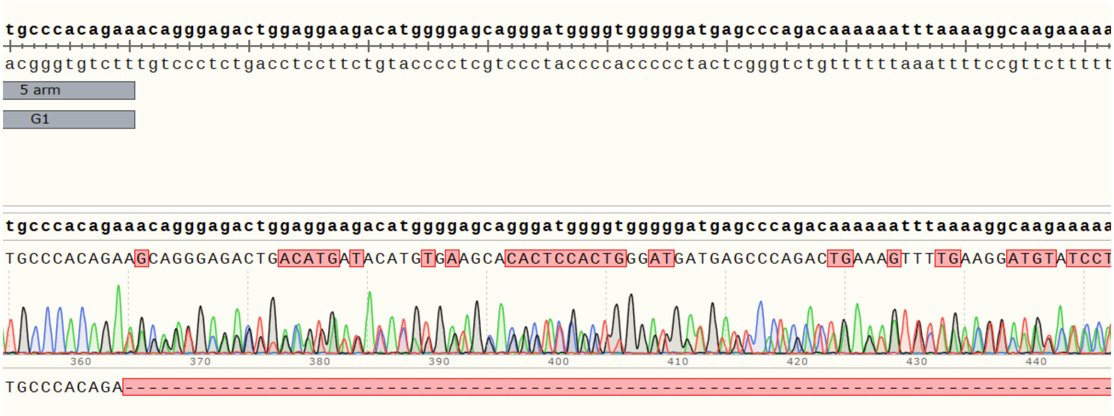

Supplementary Fig.S1 Sanger sequencing. Sanger sequencing: the double peaks shown in the figure represent HE.

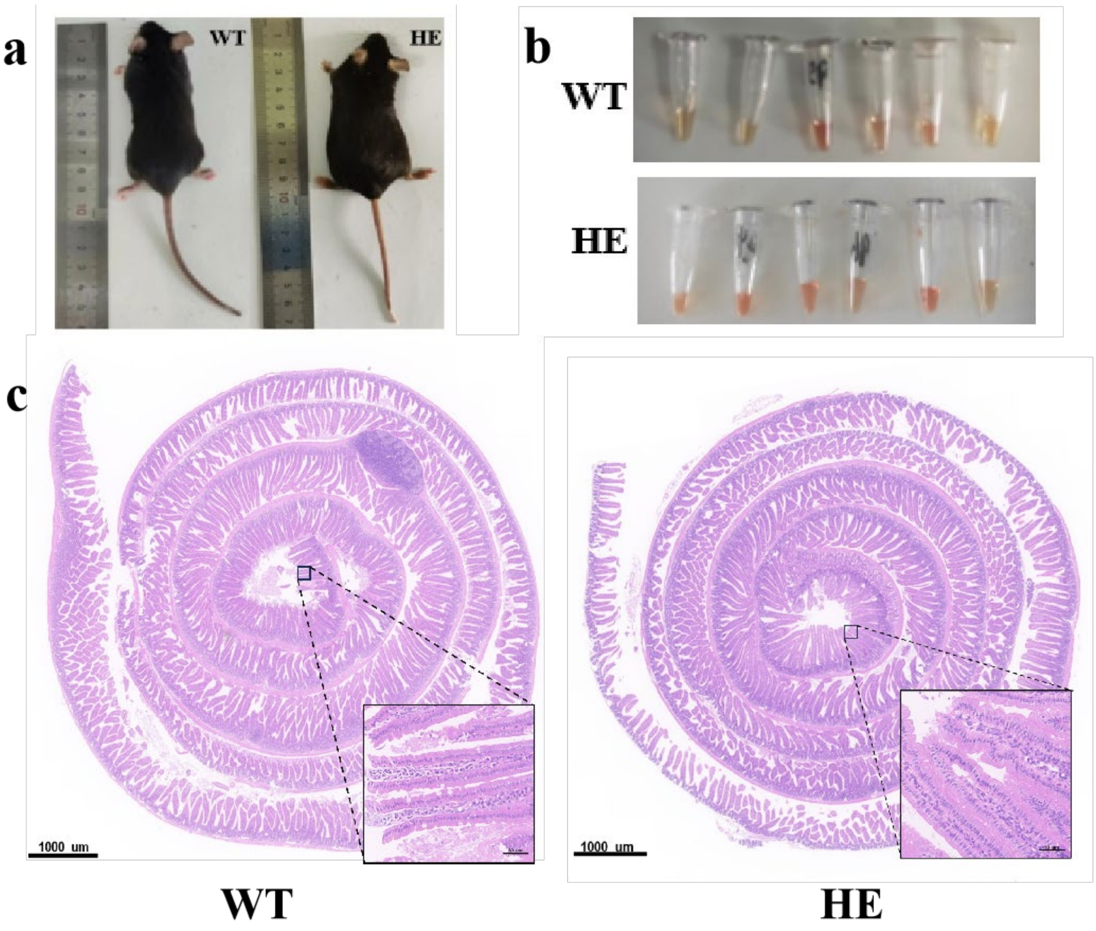

Supplementary Fig.S2 Comparison of phenotypes. Appearance of the mice(a)and serum(b) (N = 6); (c) Histomorphological findings of intestinal lipid deposition, H&E staining of intestinal tissues.

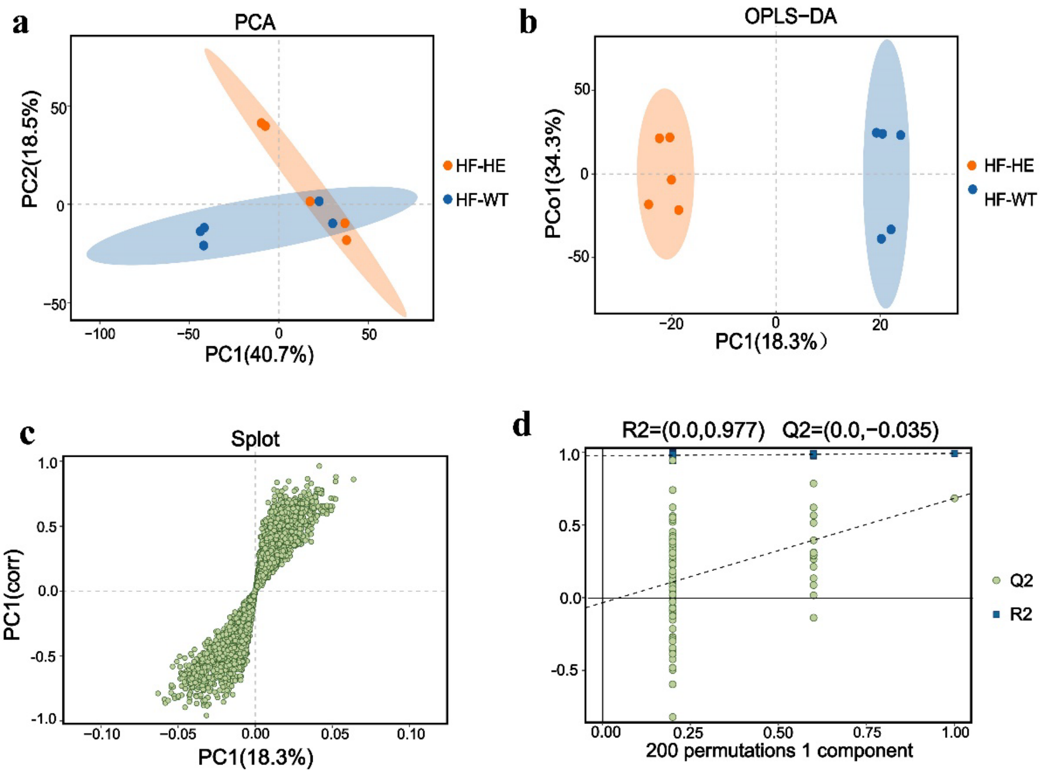

Supplementary Fig.S3 Overview and multivariate statistical analysis. (a) PCA score chart. (b) Score plot analysis of OPLS-DA of group of HF-HE and hf-WT. Each point corresponds to a sample. Different groups are marked with different colors, and the region marked by ellipse is the 95% confidence region of sample points (N = 5, respectively). (c) The figure of the Splot. The horizontal axis of the Splot graph represents the characteristic values of the influence of metabolites on the comparison group, and the vertical axis shows the correlation between the sample score and the metabolites. Since the eigenvalues and correlations are both positive and negative, all the points in the visualized graph are distributed in the first and third quadrants, similar to an S shape, which is called Splot. (d) The fitted graph was detected. All the green Q2 values on the left are lower than the original points on the right, indicating that the original model did not overfit.

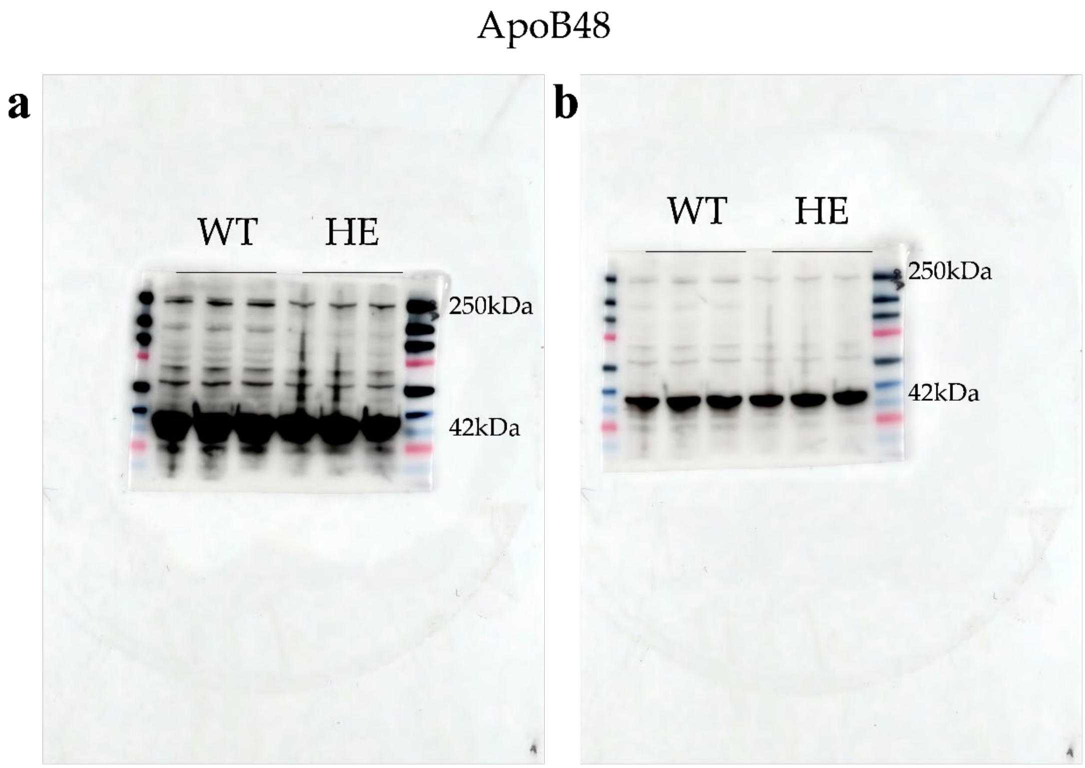

Supplementary Fig.S4 Original Western blot images corresponding to Figure 1(g) in the main text. Protein expression of ApoB48 (~250 kDa) with β-actin (~42 kDa). Molecular weight markers are indicated. (a) The exposure time of chemiluminescence is long. (b) The exposure time of chemiluminescence is short.

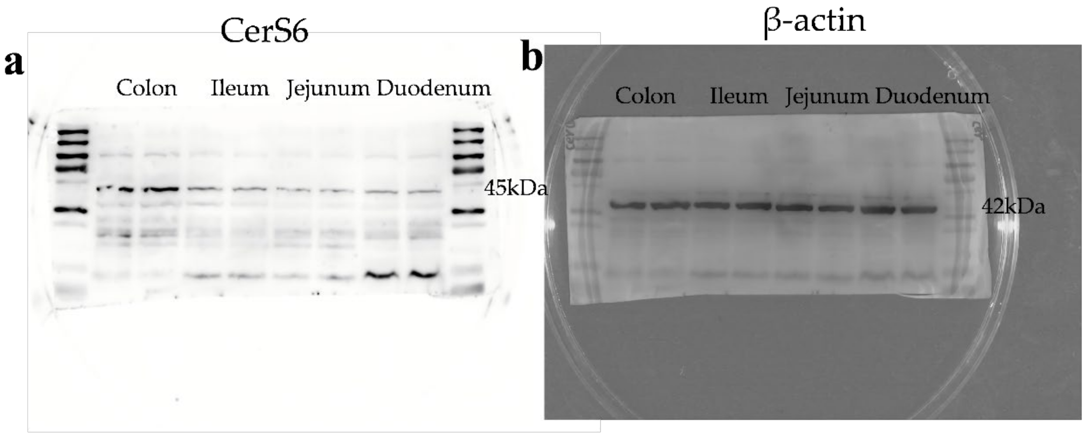

Supplementary Fig.S5 Original Western blot images corresponding to Figure 10 (c) in the main text. Protein expression of (a) CerS6 (~45 kDa) with (b) β-actin (~42 kDa). Molecular weight markers are indicated. Because the molecular weights are close, after the CerS6 protein is eluted, β-actin protein is chemiluminescent again on the same membrane.

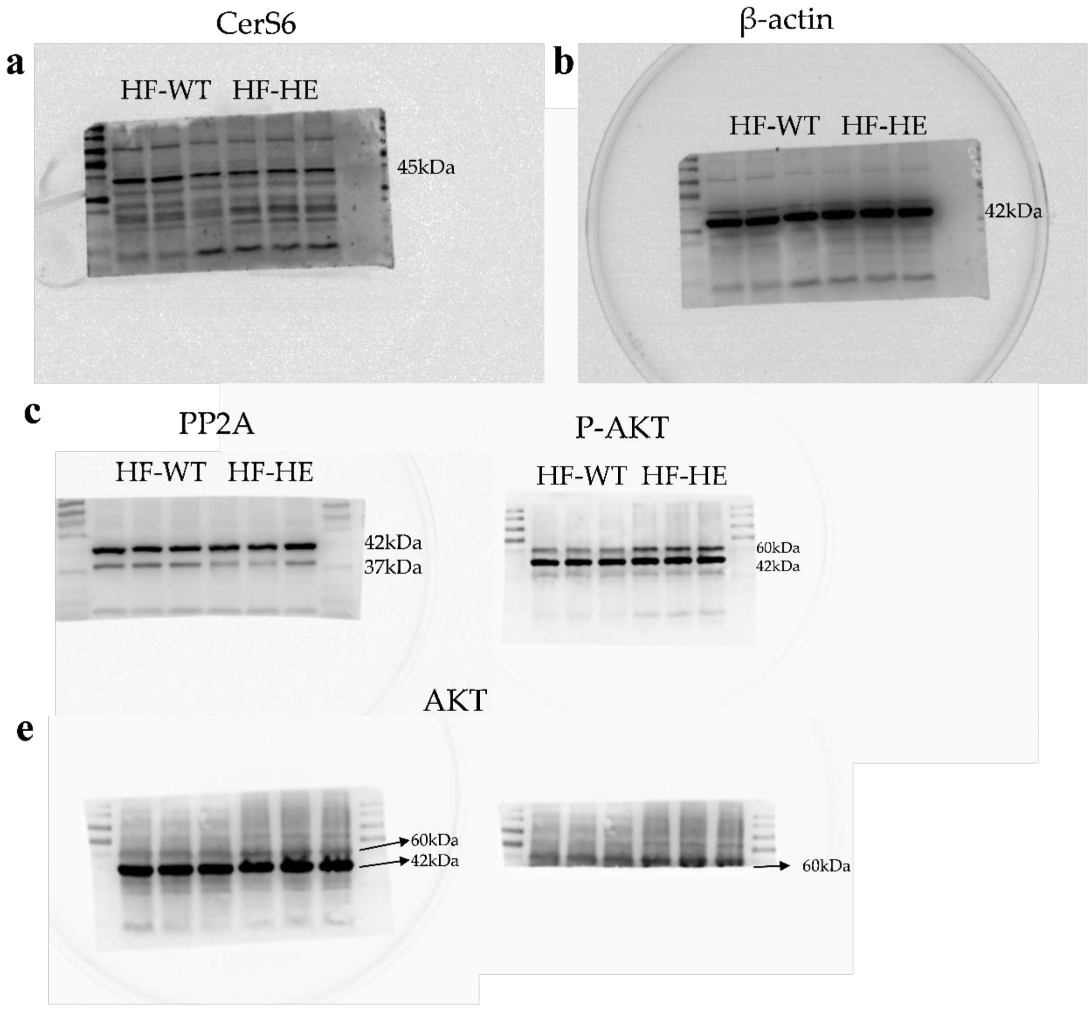

Supplementary Fig.S6 Original Western blot images corresponding to Figure 10 (d) in the main text. Protein expression of (a) CerS6 (~45 kDa) with (b) β-actin (~42 kDa). Because the molecular weights are close, after the CerS6 protein is eluted, β-actin protein is chemiluminescent again on the same membrane. (c) Protein expression of PP2A (~37 kDa) with β-actin (~42 kDa). (d) Protein expression of P-AKT (~60 kDa) with β-actin (~42 kDa). (e) Protein expression of AKT (~60 kDa) with β-actin (~42 kDa).
